# Case Report: Complete response and prolonged survival to alectinib in a NSCLC patient with metastases harboring a rare *WDR43::ALK* structural deletion

**DOI:** 10.3389/fonc.2026.1886919

**Published:** 2026-08-06

**Authors:** Lauren Epler, Giovanny Velasquez, Isa Mambetsariev, Javier Arias-Romero, Jeremy Fricke, Neil Chen, Razmig Babikian, Linh Ngo, Leonidas Arvanitis, Amit A. Kulkarni, Deniz A. Urgun, Stephen Gruber, Michelle Afkhami, Ravi Salgia

**Affiliations:** 1 Department of Medical Oncology and Therapeutics Research, City of Hope Comprehensive Cancer Center, Duarte, CA, United States; 2 Department of Biology, Northwestern University, Chicago, IL, United States; 3 Department of Pharmacy, City of Hope Comprehensive Cancer Center, Duarte, CA, United States; 4 Department of Pathology, City of Hope Comprehensive Cancer Center, Duarte, CA, United States; 5 Department of Medical Oncology and Therapeutics Research, City of Hope Phoenix, Goodyear, AZ, United States; 6 Department of Diagnostic Radiology, City of Hope Comprehensive Cancer Center, Duarte, CA, United States

**Keywords:** *ALK* structural deletion, *ALK* tyrosine kinase inhibitor, NSCLC, precision oncology, *WDR43::ALK*

## Abstract

Anaplastic lymphoma kinase (*ALK*) rearrangements define a distinct molecular subset of non–small cell lung cancer (NSCLC) that is highly sensitive to *ALK* tyrosine kinase inhibitors (TKIs). While canonical EML4::*ALK* fusions are well characterized, the clinical significance of rare noncanonical ALK rearrangements and structural alterations remains incompletely understood and may present interpretive challenges across sequencing platforms. Here, we present the case of a 61-year-old never-smoker with metastatic lung adenocarcinoma harboring a rare *ALK* structural deletion identified through complementary genomic profiling. Initial comprehensive genomic profiling identified an EML4::*ALK* rearrangement, while subsequent orthogonal DNA/RNA genomic based profiling identified a rare WDR43::ALK structural deletion resulting in a fusion transcript. ALK immunohistochemistry demonstrated ALK protein expression, supporting oncogenic *ALK* pathway activation despite the atypical genomic architecture. The patient was treated with frontline alectinib and achieved a rapid, deep, and durable response with sustained systemic and intracranial disease control for more than six years. This case highlights the importance of integrative genomic interpretation in precision oncology and supports that rare ALK structural alterations may retain clinically meaningful sensitivity to modern ALK inhibition. As comprehensive sequencing becomes increasingly incorporated into routine oncologic practice, correlation of genomic findings with protein expression, clinicopathologic features, and treatment response may help determine the therapeutic relevance of unconventional *ALK* alterations.

## Introduction

Anaplastic lymphoma kinase (*ALK*) gene rearrangements define a distinct molecular subset of non–small cell lung cancer (NSCLC), most frequently involving fusion of the echinoderm microtubule-associated protein-like 4 (*EML4*) gene with *ALK* and occurring in approximately 3–5% of NSCLC cases. These rearrangements predict sensitivity to *ALK* tyrosine kinase inhibitors (TKIs) such as crizotinib and second-generation inhibitors like alectinib, which have significantly improved outcomes in advanced *ALK*-positive disease (1). Beyond canonical *EML4*–*ALK* fusions, a growing spectrum of noncanonical *ALK* rearrangements and rare structural variants has been identified in NSCLC and other malignancies. Because many of these alterations are rare, their detection and characterization may vary across DNA- and RNA-based next-generation sequencing (NGS) platforms, posing interpretive challenges in clinical practice (2).

Although numerous *ALK* fusion partners have been described, the clinical relevance of rare structural deletions involving *ALK*, including their functional relevance and responsiveness to modern *ALK* inhibitors, remains poorly characterized. Case reports and small series suggest that select noncanonical *ALK* alterations may retain sensitivity to approved *ALK*-directed therapies such as crizotinib and alectinib (3). As comprehensive genomic profiling becomes increasingly integrated into routine oncologic care, clinicians are frequently confronted with rare or atypical *ALK* alterations that fall outside well-characterized fusion patterns. Such findings are often annotated differently across sequencing platforms and incompletely represented in existing variant databases, complicating therapeutic decision-making. In this setting, integration of genomic data with protein expression, clinicopathologic features, and treatment response is often required to infer oncogenic relevance and guide targeted therapy selection (4).

Here, we report a case of metastatic lung adenocarcinoma harboring a rare *ALK* structural deletion (*WDR43*::*ALK*) identified through complementary genomic profiling, associated with a rapid, deep, and durable response to frontline alectinib. To our knowledge this is the first reported clinical finding of the *WDR43::ALK* structural deletion. The *WDR43* (WD Repeat-Containing Protein) gene, also known as UTP5 and NET12, is a protein coding gene which is a ribosome biogenesis factor that coordinates hyperactive transcription and ribogenesis (5, 6). ALK immunohistochemistry demonstrated ALK protein expression, supporting oncogenic *ALK* pathway activation despite the atypical genomic architecture. This case highlights the importance of integrative genomic interpretation in precision oncology and adds to the limited clinical evidence supporting clinically actionable *ALK* alterations.

## Case presentation

**Figure 1:**
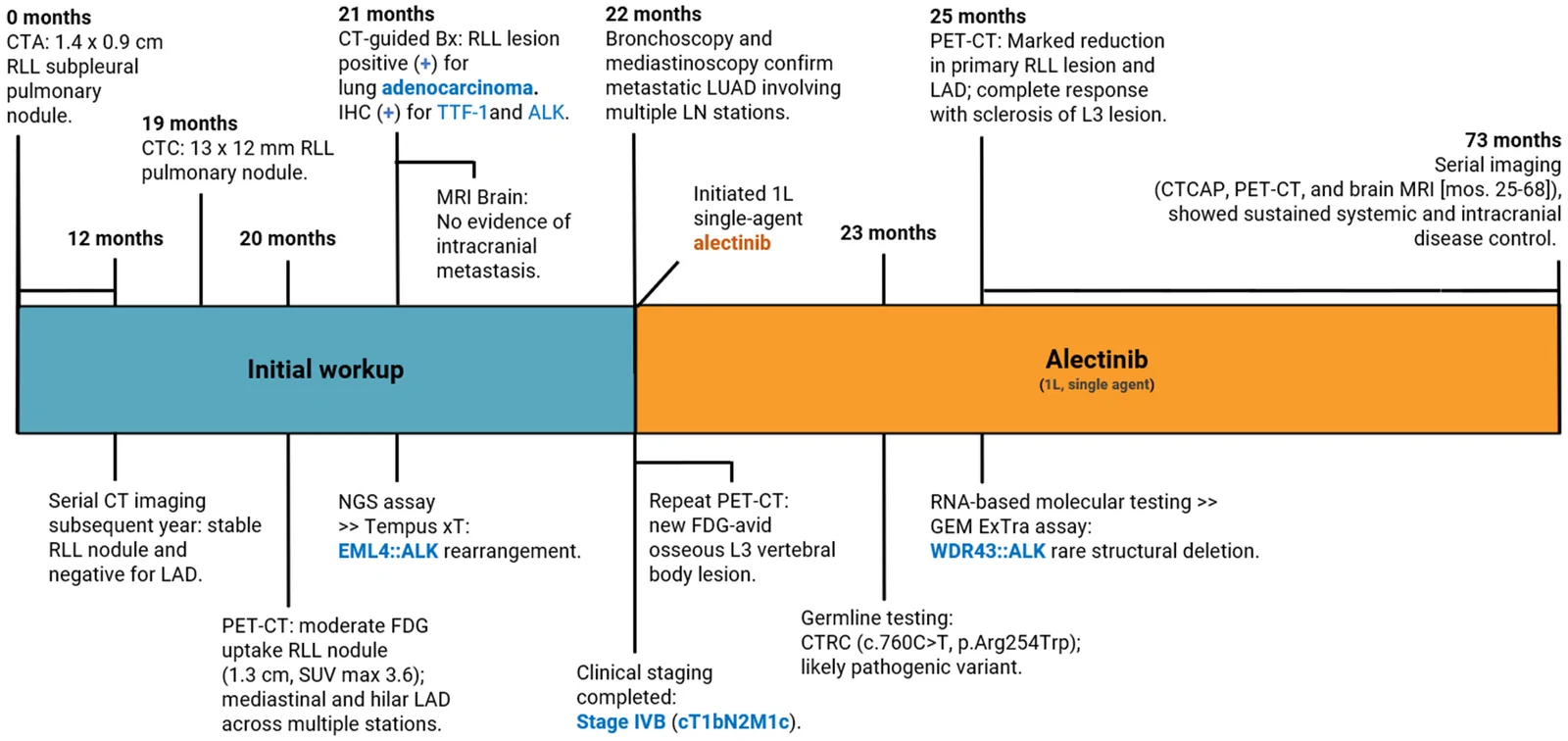
Patient clinical management timeline. Months represent de-identified endpoints for each of the events in relation to the initial scan finding.

A 61-year-old male never-smoker with a history of coronary artery disease, cardiomyopathy, hypertension, gastroesophageal reflux disease (GERD), benign prostatic hyperplasia (BPH), and hyperlipidemia underwent coronary computed tomography angiography (CTA) initially for cardiac evaluation. Family history was notable for leukemia in a brother and myocardial infarction in both his father and sister. The CTA incidentally revealed a spiculated subpleural pulmonary nodule in the posterior right lower lobe (RLL) measuring 1.4 × 0.9 cm, suspicious for primary lung malignancy, without associated lymphadenopathy (month 0; **Figure 1**). A repeat contrast-enhanced CT chest several months later confirmed persistence of the RLL nodule. Surveillance was delayed due to intervening medical events, including orthopedic surgery and COVID-19 infection. Serial CT imaging over the subsequent year demonstrated stability of the RLL nodule without interval growth or lymphadenopathy. Although the nodule remained radiographically stable, its persistent spiculated appearance continued to raise concern for an underlying primary lung malignancy. Therefore, positron emission tomography–computed tomography (PET-CT) was obtained for further characterization and staging.

Nineteen months after initial CTA evaluation, a non-contrast CT chest demonstrated a 13x12 mm solid pulmonary nodule in the right lower lobe (RLL). PET-CT at 20 months demonstrated moderate fluorodeoxyglucose (FDG) uptake in the RLL nodule (1.3 cm; SUV max 3.6) as well as FDG-avid mediastinal and hilar lymphadenopathy involving multiple nodal stations. CT-guided biopsy of the RLL lesion at 21 months confirmed lung adenocarcinoma with an acinar growth pattern. His ECOG at diagnosis was 0, indicating that he was fully active without restriction. Immunohistochemistry demonstrated tumor cell positivity for thyroid transcription factor-1 (TTF-1) and *ALK (clone 54A*, performed at an outside reference laboratory, UCLA). Programmed death-ligand 1 (PD-L1) expression was low (tumor proportion score 5%). Targeted molecular testing using the Idylla platform was negative for EGFR mutations.

Brain magnetic resonance imaging at 21 months showed no intracranial metastases. Around the same time, comprehensive tumor profiling using the Tempus xT assay (648-gene DNA and RNA panel) identified an EML4::ALK rearrangement with pathogenic co-occurring alterations in TP53 (R110L) and TRAF7 (c.1135 + 2_1135 + 3del, splice site). A WDR43::ALK structural alteration was not identified by the Tempus xT assay despite coverage of the WDR43 locus. Tumor mutational burden was low (3.2 mutations/Mb), and microsatellite instability testing demonstrated a stable profile. Mediastinal staging workup with bronchoscopy and mediastinoscopy at 22 months confirmed metastatic adenocarcinoma in multiple mediastinal lymph node stations. This prompted the patient’s enrollment in City of Hope’s (COH) institutional IRB-approved protocol for germline testing, as well as additional molecular profiling using the Ashion GEM ExTra whole-exome DNA and RNA sequencing assay. Repeat PET-CT performed the same month demonstrated a new FDG-avid osseous lesion in the L3 vertebral body, consistent with metastatic disease. The patient was diagnosed with stage IVB (cT1b cN2 cM1c) lung adenocarcinoma according to the American Joint Committee on Cancer (AJCC), 8th edition, staging system.

**Figure 2:**
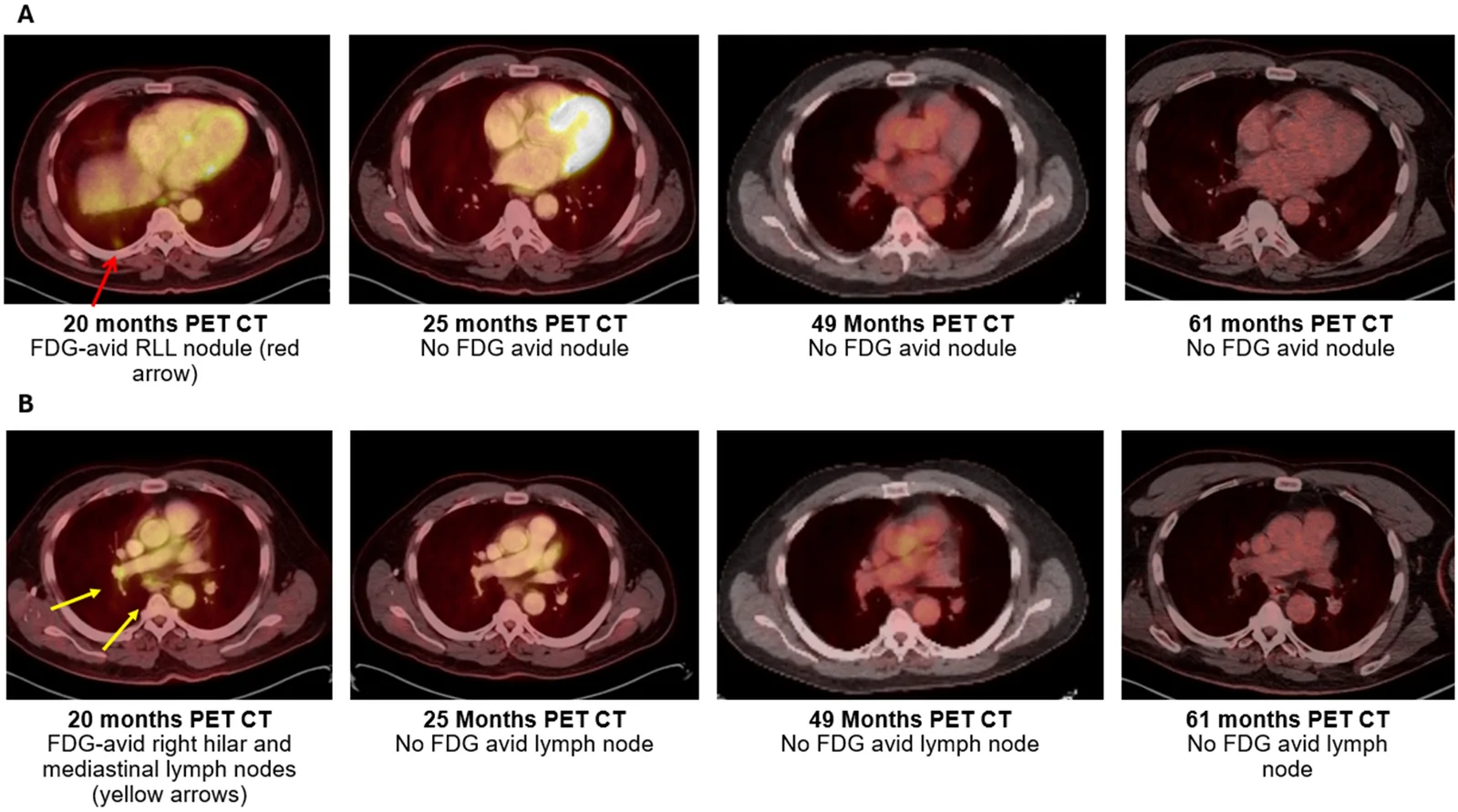
PET CT radiological imaging over time. **(A)** Presentation and resolution of an FDG-avid RLL nodule over the treatment period. **(B)** Presentation and resolution of FDG-avid right hilar and mediastinal lymph nodes during alectinib treatment.

**Figure 3:**
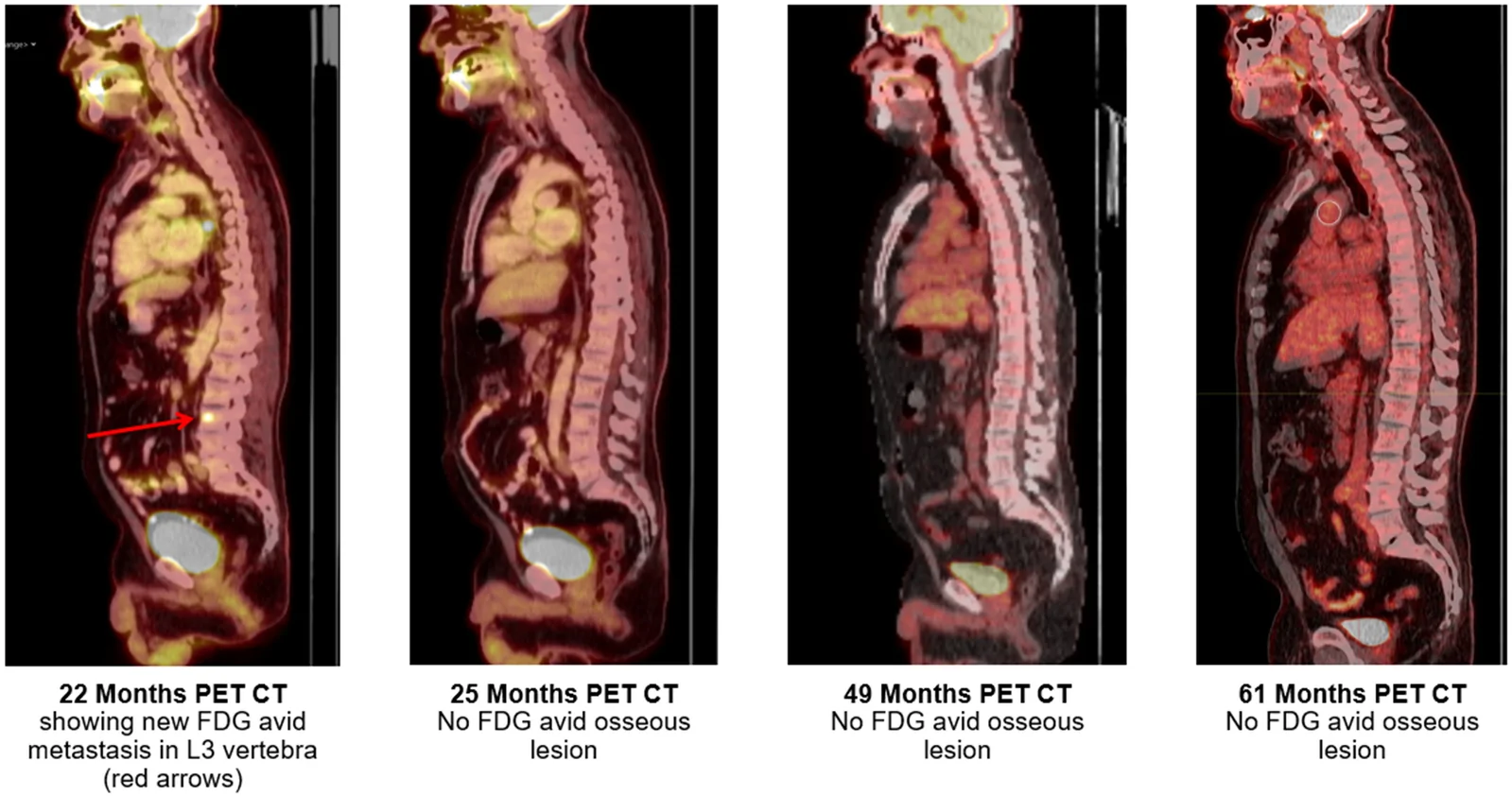
Sagittal view of PET CT demonstrating the FDG avid metastasis in L3 vertebra followed by metabolic resolution and sclerosis of the L3 lesion.

Frontline targeted therapy with alectinib (600 mg orally twice daily) was initiated at 22 months following the initial CTA evaluation. His ECOG performance status at treatment initiation was 1 with a reported cough and back and neck pain. Alectinib was tolerated well without treatment-related toxicity during follow-up, and the patient reported improved breathing and the ability to exercise multiple times a week. At month 23, germline testing performed under an institutional IRB-approved protocol identified a heterozygous likely pathogenic CTRC c.760C>T (p.Arg254Trp) variant associated with chronic pancreatitis risk, along with variants of uncertain significance in *ERBB2, FANCE*, and *SLX4*. The germline findings were incidental and did not alter clinical management. Early response assessment at 25 months demonstrated marked reduction of the primary RLL lesion (**Figure 2A**) and mediastinal lymphadenopathy (**Figure 2B**). Follow-up PET-CT showed complete metabolic resolution of pulmonary and nodal disease with sclerosis of the L3 lesion, consistent with treatment response of the osseous metastasis (**Figure 3**).

**Figure 4:**
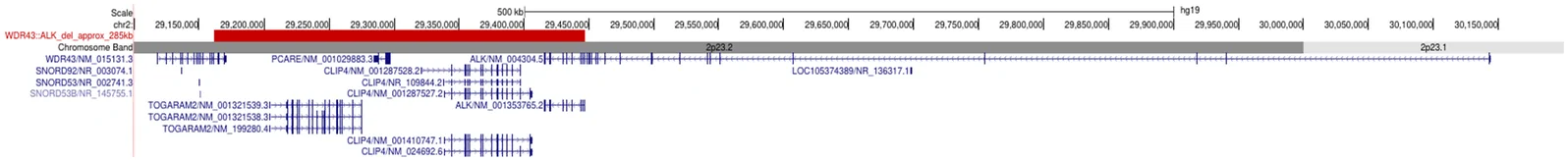
Chromosomal schematic of WDR43::ALK structural deletion at 2p23 locus, GRCh37/hg19, UCSC Genome Browser (http://genome.ucsc.edu).

Notably at 25 months, additional RNA-based molecular testing of mediastinal lymph node tissue using the GEM ExTra assay (Exact Sciences) identified a rare *ALK* structural deletion (WDR43::*ALK*) of DNA origin located at coordinates chr2:29161690; chr2:29446729 (**Figure 4**). The previously identified EML4::ALK rearrangement was not reported on this assay, prompting further integrative genomic interpretation to reconcile the discrepant molecular findings. Surveillance imaging between months 25 and 68, including CT of the chest, abdomen, and pelvis and serial brain MRI, demonstrated sustained systemic disease control without evidence of intracranial progression (**Figures 2**, **3**). The patient remains on alectinib with ongoing clinical and radiographic response at 52 months after pathology-confirmed diagnosis and 51 months after initiation of alectinib (73 months after the initial CTA evaluation). His ECOG status at last follow-up was 1, with mild fatigue. No other treatment-related toxicities were reported.

## Discussion

This case illustrates that a rare *ALK* structural deletion (*WDR43::ALK*) can function as a clinically actionable oncogenic driver in metastatic lung adenocarcinoma. The patient experienced a rapid, deep, and durable response to frontline alectinib, with sustained systemic and intracranial disease control over several years. These findings support preserved *ALK* dependency despite a noncanonical genomic configuration and add to the limited clinical evidence supporting *ALK* alterations associated with meaningful clinical benefit from *ALK*-directed therapy.

Canonical *EML4*::*ALK* fusions drive oncogenesis through constitutive activation of the *ALK* kinase domain and are well-established predictors of sensitivity to *ALK* tyrosine kinase inhibitors. Emerging evidence from large-scale genomic profiling studies indicates that atypical ALK rearrangements may retain oncogenic potential when the ALK kinase domain is preserved (7). More recent case reports have also demonstrated durable responses to alectinib in patients harboring uncommon ALK fusion partners, supporting the therapeutic relevance of selected noncanonical ALK rearrangements. Published clinical experience with WDR43::ALK remains limited, and its biological behavior and expected sensitivity to ALK inhibition are not yet well defined. To place the present case in context, **Table 1** provides a non-exhaustive summary of published noncanonical and rare ALK rearrangements in NSCLC, specifically highlighting detection methods, ALK-directed therapies, and clinical outcomes. In this case, ALK protein expression and prolonged sensitivity to alectinib suggest that the rare WDR43::ALK structural deletion is functionally relevant and sufficient to confer ALK dependence despite its noncanonical architecture.

**Table 1:** Representative rare and noncanonical ALK rearrangements in NSCLC.

ALK rearrangement	Detection method	ALK TKI	Reported best response	PFS/duration of response	References
STRN::ALK	Targeted NGS	Alectinib	Excellent response	>19 mo	Su et al., 2020 (9)
STRN::ALK	ALK IHC → NGS	Alectinib → Crizotinib	Limited clinical response (co-occurring MET amp)	PFS 4 mo (alectinib); PFS 11 mo (crizotinib)	Li et al., 2021 (10)
KIF5B::ALK	NGS	Lorlatinib	Partial response	Durable (ongoing at report)	Luo & Ouyang, 2025 (11)
HIP1::ALK	NGS	Crizotinib; Lorlatinib	ORR 90% (crizotinib cohort); Partial response reported with lorlatinib	Median PFS 17.9 mo (crizotinib); DOR >26.5 mo (lorlatinib)	Kang et al., 2022 (12)
GCC2::ALK	DNA-based NGS with confirmatory sequencing	Crizotinib → Ceritinib	Clinical benefit (crizotinib); Partial response (ceritinib)	PFS 18 mo (crizotinib); PR ongoing (ceritinib)	Jiang et al., 2018 (13)
LMO7::ALK	ALK IHC → NGS	Alectinib → Ensartinib	Primary resistance (alectinib); Clinical benefit (ensartinib)	PFS 18 mo (ensartinib)	Li et al., 2021 (10)
CLIP1::ALK	RNA-based NGS	Crizotinib†	Clinical benefit reported †	Not individually reported†	Vendrell et al., 2017 (14)
WDR43::ALK (Present Case)	Complementary genomic profiling (Tempus xT and GEM ExTra)	Alectinib	Complete response	PFS >51 mo (ongoing)	Present case

Summary of published cases and series reporting rare and noncanonical ALK rearrangements in NSCLC, including detection methodology, ALK TKI therapy, best response, and clinical outcomes. STRN::ALK is presented in two separate rows to illustrate divergent clinical outcomes reported in the literature, including one case with concurrent MET amplification. Response categories reflect the terminology used in each cited publication and may not uniformly correspond to formal RECIST criteria.

ALK, anaplastic lymphoma kinase; CR, complete response; IHC, immunohistochemistry; MET amp, MET amplification; mo, months; NGS, next-generation sequencing; NSCLC, non-small cell lung cancer; ORR, objective response rate; PFS, progression-free survival; TKI, tyrosine kinase inhibitor.

† CLIP1::ALK was identified in addition to DCTN1::ALK and GCC2::ALK as novel ALK rearrangements. Two of three patients harboring these novel fusions were treated with and sensitive to crizotinib; individual patient-level outcomes were not reported separately for each fusion.

This case also highlights the challenges associated with interpreting uncommon ALK alterations across different genomic platforms. In this case, two comprehensive genomic profiling assays may yield discrepant variant results because of underlying tumor heterogeneity, differences in assay design, sequencing coverage, and bioinformatic interpretation, rather than technical error (4). In this patient, the Tempus xT assay identified a canonical *EML4*–*ALK* rearrangement, while complementary whole-exome DNA and RNA sequencing using the GEM ExTra assay detected a rare WDR43::ALK structural alteration. Together, these findings underscore the importance of utilizing complementary genomic approaches to fully characterize atypical *ALK* rearrangements when the underlying genomic architecture is complex.

In cases involving complex genomic architecture or atypical structural alterations, clinical response to targeted therapy may serve as a surrogate marker of dependence on an oncogenic pathway. This patient’s sustained systemic and intracranial disease control following initiation of alectinib provides compelling clinical evidence that ALK signaling remained the dominant oncogenic driver.

From a molecular interpretation standpoint, this case illustrates the limitations of relying solely on canonical fusion architecture to infer oncogenic relevance. Rare or noncanonical structural alterations are often underrepresented in existing variant databases, and their functional consequences are frequently inferred indirectly based on predicted protein impact, including preservation of the kinase domain, rather than direct experimental validation (8). The sustained *ALK* inhibitor sensitivity observed in this case supports the concept that preservation of *ALK* kinase activity, rather than strict adherence to known fusion configurations, may be sufficient to confer oncogenic dependency. This report has several limitations. The molecular findings were generated using commercially available clinical sequencing assays, and raw sequencing data were not available for independent review. Consequently, the precise genomic architecture underlying this alteration could not be fully characterized. Although both Tempus xT and GEM ExTra incorporate DNA and RNA sequencing, differences in assay design, sequencing coverage, bioinformatic pipelines, and variant interpretation may have contributed to the discrepant molecular findings. Additional studies will be needed to better understand the clinical significance of rare ALK structural alterations and their interpretation across complementary sequencing platforms.

In conclusion, this case supports the concept that, as sequencing becomes increasingly comprehensive and rare structural variants continue to be identified, reliance on canonical fusion patterns alone may be insufficient. This report supports a pragmatic approach in which genomic findings are interpreted alongside protein expression, clinicopathologic features, and longitudinal treatment response to guide therapeutic decision-making. As the catalog of noncanonical *ALK* alterations continues to expand, systematic aggregation of similar cases and complementary functional studies will be essential to refine diagnostic frameworks and ensure that patients with actionable but unconventional alterations are not excluded from effective targeted therapies.

## Data Availability

The original contributions presented in the study are included in the article/supplementary material. Further inquiries can be directed to the corresponding author.
