# Correction: Case Report: Complete response and prolonged survival to alectinib in a NSCLC patient with metastases harboring a rare WDR43::ALK structural deletion

**DOI:** 10.3389/fonc.2026.1961716

**Published:** 2026-09-11

**Authors:** Frontiers Production Office

**Affiliations:** Frontiers Media SA, Lausanne, Switzerland

**Keywords:** *ALK* structural deletion, *ALK* tyrosine kinase inhibitor, NSCLC, precision oncology, *WDR43::ALK*

There was a mistake in **Figure 1** as published. EML4::ALK was erroneously written as ML4::ALK. The corrected **Figure 1** appears below.

There was a mistake in **Figure 3** as published. **Figure 3** was erroneously replaced by a duplicate of **Figure 1**. The corrected **Figure 3** appears below.

The original version of this article has been updated.

**Figure 1:**
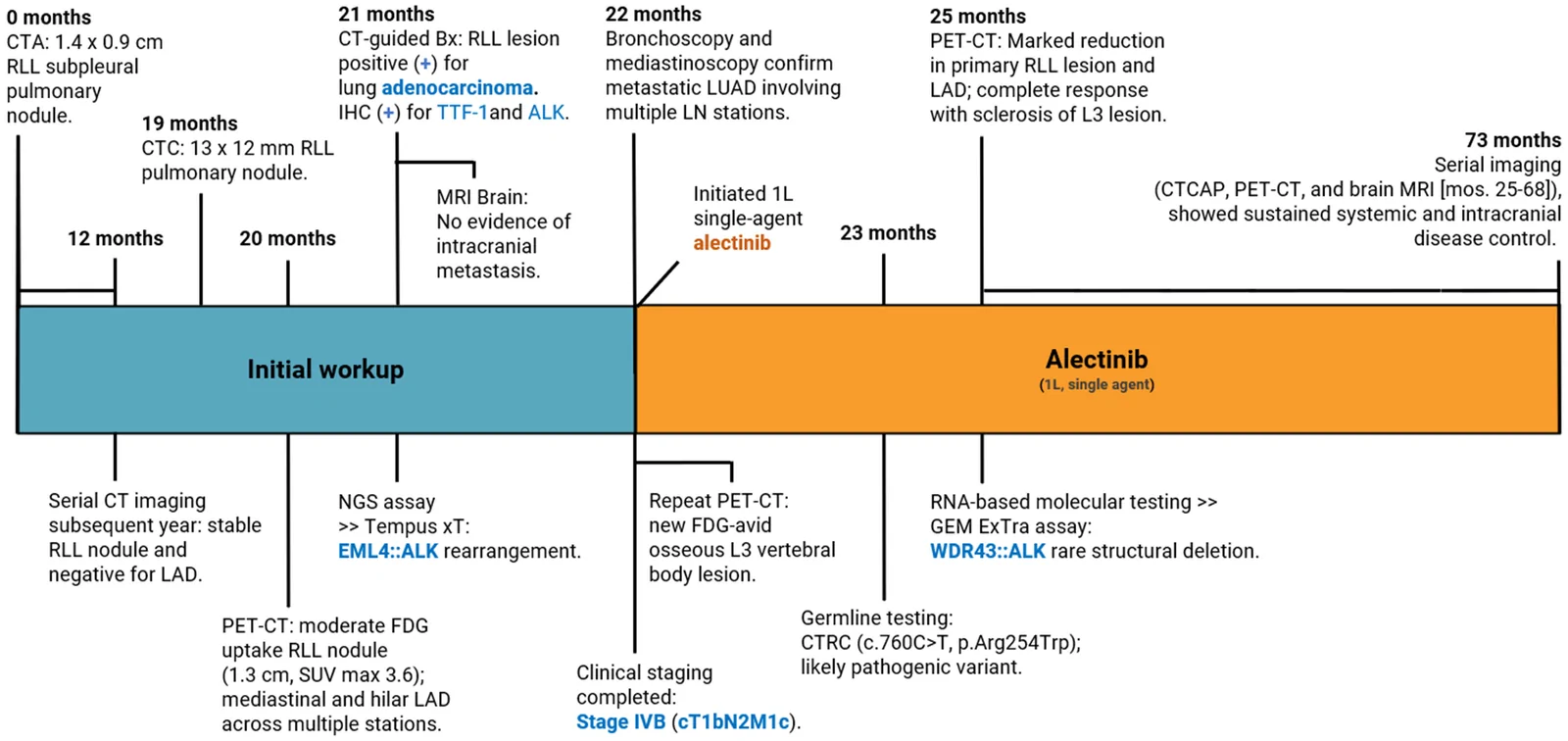
Patient clinical management timeline. Months represent de-identified endpoints for each of the events in relation to the initial scan finding.

**Figure 3:**
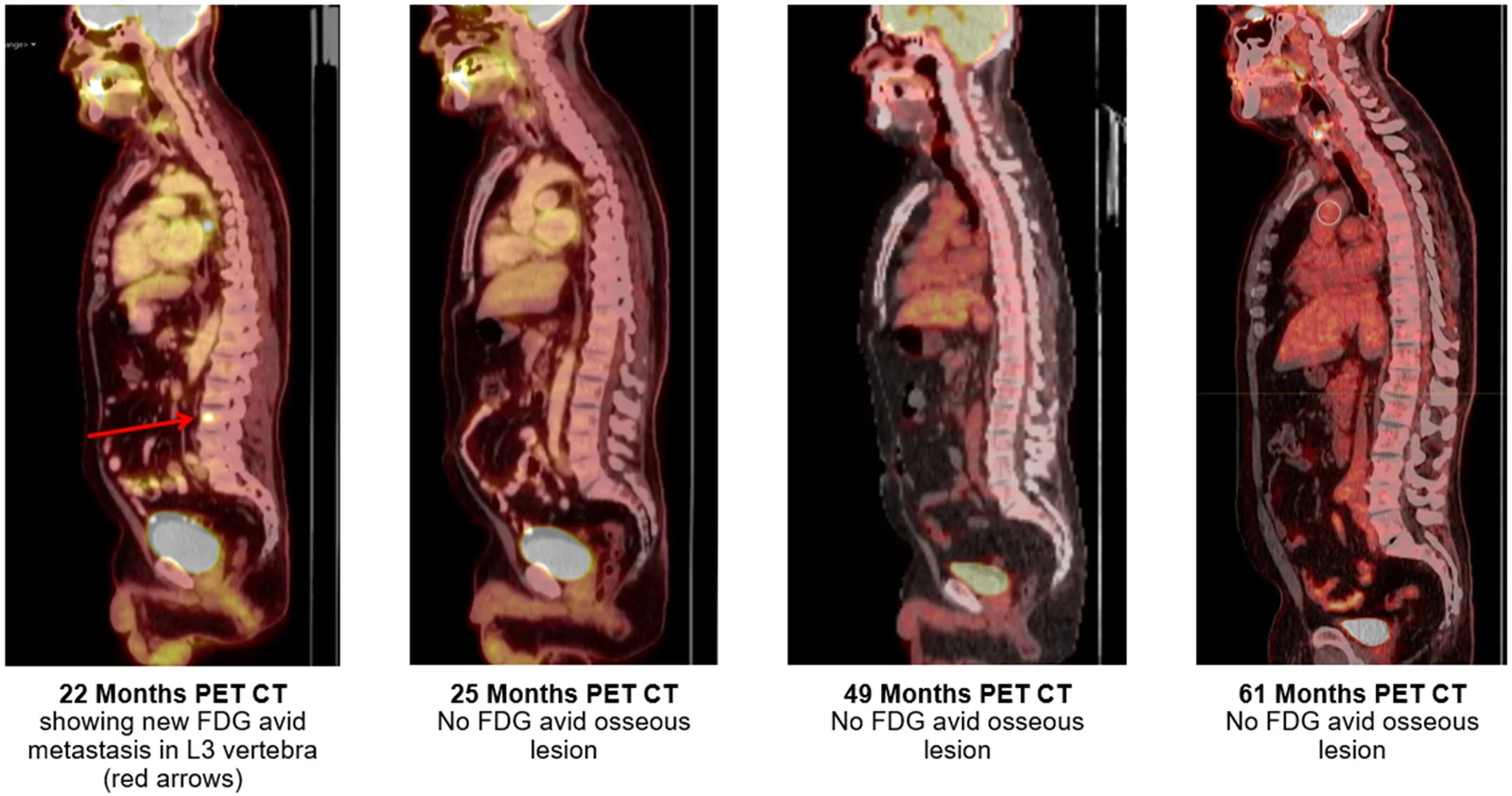
Sagittal view of PET CT demonstrating the FDG avid metastasis in L3 vertebra followed by metabolic resolution and sclerosis of the L3 lesion.

